# Social cognition abilities in patients with primary and secondary chronic pain

**DOI:** 10.3389/fpsyg.2024.1315682

**Published:** 2024-02-27

**Authors:** Alessandra Telesca, Alessandra Vergallito, Monica Consonni, Giulia Mattavelli, Alessia Ferrario, Licia Grazzi, Susanna Usai, Leonor Josefina Romero Lauro

**Affiliations:** ^1^Ph.D. Program in Neuroscience, School of Medicine and Surgery, University of Milano-Bicocca, Monza, Italy; ^2^Fondazione IRCCS Istituto Neurologico “Carlo Besta”, Neuroalgology Unit, Milan, Italy; ^3^Department of Psychology and NeuroMi, University of Milano-Bicocca, Milan, Italy; ^4^ICoN Center, Scuola Universitaria Superiore IUSS, Pavia, Italy; ^5^Istituti Clinici Scientifici Maugeri IRCCS, Cognitive Neuroscience Laboratory of Pavia Institute, Pavia, Italy

**Keywords:** chronic primary pain, chronic secondary pain, emotion recognition, social cognition, cognitive impairment, Ekman task, Story-Based Empathy Task

## Abstract

Previous evidence suggested that chronic pain is characterized by cognitive deficits, particularly in the social cognition domain. Recently, a new chronic pain classification has been proposed distinguishing chronic primary pain (CPP), in which pain is the primary cause of patients’ disease, and chronic secondary pain (CSP), in which pain is secondary to an underlying illness. The present study aimed at investigating social cognition profiles in the two disorders. We included 38 CPP, 43 CSP patients, and 41 healthy controls (HC). Social cognition was assessed with the Ekman-60 faces test (Ekman-60F) and the Story-Based Empathy Task (SET), whereas global cognitive functioning was measured with the Montreal Cognitive Assessment (MoCA). Pain and mood symptoms, coping strategies, and alexithymia were also evaluated. Correlations among clinical pain-related measures, cognitive performance, and psychopathological features were investigated. Results suggested that CSP patients were impaired compared to CPP and HC in social cognition abilities, while CPP and HC performance was not statistically different. Pain intensity and illness duration did not correlate with cognitive performance or psychopathological measures. These findings confirmed the presence of social cognition deficits in chronic pain patients, suggesting for the first time that such impairment mainly affects CSP patients, but not CPP. We also highlighted the importance of measuring global cognitive functioning when targeting chronic pain disorders. Future research should further investigate the cognitive and psychopathological profile of CPP and CSP patients to clarify whether present findings can be generalized as disorder characteristics.

## 1 Introduction

Chronic pain is a multifaceted bio-psycho-social disease that occurs when the perception of physical pain – whether connected to actual tissue damage or not – persists for an extended period, exceeding three months (IASP, 2020). The chronic pain label includes different pathologies that may involve (i) distinct body districts, such as the head (i.e., headache), back (i.e., low back pain), or limbs (i.e., peripheral neuropathies); (ii) the entire body (i.e., fibromyalgia); or (iii) the internal organs, as the irritable bowel syndrome or the vestibulodynia.

Persistent pain significantly impacts individuals’ functional abilities and social and psychological well-being (IASP, 2020). Converging evidence suggests that patients with chronic pain exhibit impairment in cognitive functions (Peters, 2015; Higgins et al., 2018; Zheng and Wang, 2019), involving learning and memory (Duschek et al., 2013; Gil-Gouveia et al., 2015), information processing speed (Moriarty et al., 2011; Pulles and Oosterman, 2011), attention and executive functions (Apkarian et al., 2005; Attal et al., 2014; Lee et al., 2015). Moreover, several studies report chronic pain patients’ difficulties in interacting with other people and highlight their poor social functioning (van Middendorp et al., 2008; Karayannis et al., 2019; Baumgartner et al., 2023; Miller, 2023), thus stressing the need for further research on social cognition impairment in these patients.

Social cognition is a complex function that entails all the cognitive abilities implemented in social interactions, including executive functioning, information processing, perception, attention, and memory (Frith, 2008). It is considered a multifaceted construct entailing different domains: *emotion processing*, defined as the ability to identify emotions – typically by facial expressions; *theory of mind*, or the ability to understand the mental states of other people and infer their intentions and beliefs; *social perception,* or the capacity to identify social contexts, roles and rules from non-verbal indices such as voice intonation and body language*; social knowledge*, indicating the awareness of roles and rules of social situations; *attributional bias or style,* that refers to the ability to infer the causes of situations or behaviors (Green et al., 2019). In particular, emotion recognition has been largely investigated in clinical assessment and research, based on the assumption that faces are the first attentional stimulus when interacting with other people as they are considered the window of one’s inner emotions (Frith, 2008).

Previous evidence on patients affected by chronic pain highlights the presence of some deficits in social cognition (Shin et al., 2013; Szabó et al., 2019; Chaves et al., 2021; Guevara et al., 2021; Grabli et al., 2022; Raimo et al., 2022). These studies typically compare the performance between patients with specific pain diagnoses (e.g., migraine, fibromyalgia) and healthy controls. Weiß et al. (2013), for instance, administered the Karolinska Directed Emotional Faces Battery (Lundqvist et al., 1998) to assess facial emotion recognition in thirty-five patients with fibromyalgia and healthy controls, reporting poorer performances in patients than controls in recognizing facial expressions, independently from the specific emotion. Interestingly, performance decreased at higher mood symptoms and referred pain intensity, whereas the presence of psychiatric comorbidities and medication consumption (antidepressants, analgesics, anxiolytics, or opiates) had no impact on emotion recognition. Similar findings emerged in another study comparing fibromyalgia patients and healthy controls in emotion recognition and executive functions abilities (Guevara et al., 2021). The authors administered the Ekman-60 faces test (Ekman-60F) to investigate facial expression recognition and several tasks evaluating different components of executive functions, such as shifting, updating, and inhibition processes. Results revealed poorer performances in patients than healthy controls, in both social cognition and executive functioning, with positive correlations between the ability of recognizing emotions and the performance in the executive functions tasks, whereas the comorbidity with anxiety, depression, and medication consumption did not impact on Ekman-60F performance (Guevara et al., 2021).

Another study (Shin et al., 2013) investigated social cognition abilities in patients with complex regional pain syndrome by mean of the Reading Mind in the Eyes Test (RMET) (Baron-Cohen et al., 2001). Nonsocial cognitive abilities targeting psychomotor speed, attention and inhibitory processes were also evaluated. Results highlighted that patients were selectively impaired in recognizing emotional states, whereas no differences with healthy participants emerged in the nonsocial domains. Notably, patients’ RMET performance was negatively correlated to the affective dimension of pain (i.e., tiredness, sickness, fear, and punishment scores) as measured by the short form of the McGill Pain Questionnaire (Melzack, 1987). Social cognition abilities in chronic low back pain have been investigated by Grabli et al. (2022) using the facial emotion recognition and the mentalizing tasks from the mini- Social Cognition and Emotional Assessment (SEA) (mini-SEA) (Bertoux et al., 2012): results highlighted that patient had lower theory-of-mind abilities compared to healthy controls. One study focused on musculoskeletal pain patients (Chaves et al., 2021), using a modified version of the Ekman-60F test, and reported worst performances in the emotional face recognition task in patients than healthy controls (Chaves et al., 2021).

Finally, two studies explored social cognition abilities in patients with migraine (Szabó et al., 2019; Raimo et al., 2022). Raimo et al. (2022) analyzed patients’ performance on theory of mind, by administering several tasks, among which the RMET, the modified Italian version of the Emotion Attribution Task (EAT) (Blair and Cipolotti, 2000), the Theory of Mind Picture Sequencing Task (TMPS) (Brüne, 2003) and the Italian version of the Advanced Test of ToM (ATT) (Prior et al., 2003). Results showed a worse performance in migraine patients compared with healthy controls in all tasks except for the RMET, in which both populations performed similarly. Moreover, by mean of a comprehensive neuropsychological assessment that included tasks evaluating executive functioning, namely reasoning abilities and cognitive flexibility, and memory, authors found a significant relation between theory of mind abilities and both executive functioning and memory. This result suggested that good executive functioning is necessary for a good performance in social cognition (Raimo et al., 2022). Additionally, Szabó et al. (2019) used functional magnetic resonance imaging (fMRI) to measure brain activity during an implicit facial expression recognition task in which they were asked to indicate the gender of faces expressing happiness, fear, and sadness, plus neutral faces. Only in the patients’ group authors described an increased activity in right inferior, middle and superior frontal gyri during fear faces presentation, despite no group differences were reported at the behavioral level. Moreover, the authors reported positive correlations between migraine frequency and activity in the right somatosensory regions (pre- and post-central gyri, inferior parietal cortex) for fearful expressions, and in the right putamen and caudate nucleus for happy faces. Authors concluded that migraine patients showed an enhanced response to emotionally arousing stimuli, which could have potentially served as stressors or triggers for migraine attacks.

All these data on social cognition abilities in chronic pain patients involve different populations that could be grouped under the same macro-category, named “chronic primary pain” (CPP), recently introduced in the field of chronic pain by the IASP committee (Treede et al., 2015; Nicholas et al., 2019). Indeed, in 2019, a new taxonomy has been proposed to distinguish chronic pain pathologies according to the etiology causing pain, thus obtaining the CPP category, in which idiopathic chronic pain or painful ailments are the leading cause of the disease, and chronic secondary pain (CSP) category, that refers to all the other conditions where the pain is the consequence of other pathological diseases, and include diagnoses of chronic neuropathic pain, chronic cancer-related pain, chronic post-surgical or post-traumatic pain. This new classification is sustained by neuroscientific evidence supporting that under some pathologies, traditionally segregated into individual labels (e.g., fibromyalgia, migraine, chronic low back pain, complex regional pain syndrome), there may be a common peculiar central nervous system functioning that may explain the persistence of chronic pain experience (Woolf, 2011; Martucci and Mackey, 2018; Nijs et al., 2021; Treede et al., 2022). See Supplementary Figure S1 for a graphical overview of the diseases’ categorization.

To date, the taxonomy has garnered sufficient approval within the scientific community, such that it has influenced the therapeutic guidelines of the National Institute for Health and Care Excellence (NICE) (Scholz, 2019; NICE, 2021). But, due to the novelty of such classification (Nicholas et al., 2019), the distinction between CPP and CSP has not been considered in the previous literature on social cognition and chronic pain.

Despite this, we can categorize the papers cited earlier as CPP or CSP according to the clinical information provided by the authors (i.e., sample description, diagnostic and inclusionary criteria). In this retrospective classification, we observe that all, except one articles (Chaves et al., 2021), can be considered belonging to the CPP category (Shin et al., 2013; Szabó et al., 2019; Guevara et al., 2021; Grabli et al., 2022; Raimo et al., 2022). In Chaves et al. (2021), the targeted population is the musculoskeletal pain, which can be both primary and secondary. In the former case, its origins are unknown. In the latter, it originates from ongoing nociception in musculoskeletal structures and it could be triggered by many causes, such as inflammation, structural alterations, or biomechanical effects of nervous system disorders (Perrot et al., 2019). In their paper, the authors did not specify the origin of musculoskeletal pain, hence we could not classify it with certainty.

As far as we know, only two studies investigated commonalities and differences of patients classified as CPP or CSP, focusing on individuals’ demographic and psychological features, coping strategies, perceived self-efficacy, and general health (Hornemann et al., 2020; Munk et al., 2022). Hornemann et al. (2020) described that CPP patients reported a worst quality of life compared to CSPs, especially considering the domain of mental health. Conversely, CSPs exhibited a higher number of physical comorbidities and incurred in a greater healthcare expenditure. Munk et al. (2022), instead, did not find differences between CPP and CSP patients in pain catastrophism and disability, coping strategies or self-efficacy.

Since little is known about the neurocognitive or psychological specific profiles of CPP and CSP, in the present study we aim at exploring commonalities and differences between CPP and CSP patients in social cognition abilities, comparing their performance to a group of age-matched healthy controls. We measured social cognitive abilities by using a classical emotional face recognition task, namely the Ekman-60F task (Ekman and Friesen, 1976; Dodich et al., 2014), and a theory-of-mind task, namely the Story-Based Empathy Task (SET) (Dodich et al., 2015). To further characterize our sample, we assessed participants’ global cognitive efficacy and several psychological dimensions, such as mood and anxiety symptoms, catastrophism, coping strategies, and quality of life.

## 2 Materials and methods

### 2.1 Ethical considerations and sample characteristics

This cross-sectional study was approved by the Ethical Board Committee of the Fondazione IRCCS Istituto Neurologico “Carlo Besta,” Milano. Data were collected from December 2021 to February 2023.

### 2.2 Participants

We recruited 122 Italian native-speaker participants (41 males, mean age = 51.8, SD ± 11.5) aged between 18 and 75 years old. Consecutive patients with chronic pain were diagnosed by expert neurologists (L.G., S.U.) as CPP or CSP according to the IASP criteria (Nicholas et al., 2019) during their medical appointments at the Neuroalgology Unit of the Fondazione IRCCS Istituto Neurologico “Carlo Besta” in Milano (Italy). The CPP group included 38 patients (7 males, mean age = 50.9, SD = 12.2). The CSP group comprised 43 patients (23 males, mean age = 51.8, SD = 12.2). Moreover, 41 healthy participants recruited from the general population (HC) (11 males, mean age = 52.5, SD = 10.4) were included as a control group.

Exclusion criteria for the three groups were: comorbidities with neurological conditions affecting cognition (e.g., head trauma, hydrocephalus, cerebrovascular disease), drug or alcohol abuse, neurocognitive developmental disorders, primary psychiatric disorders (e.g., bipolar disorder, major depression), dementia. Table 1 summarizes the sociodemographic and pain-related clinical data of the three groups. Patients’ specific diagnoses are reported in the Supplementary materials (Section A).

**Table 1:** Demographical and pain-related clinical data in CSP, CPP, and HC.

	No.	CPP Mean (SD)	CSP Mean (SD)	HC Mean (SD)	Group comparison (*p* value)	Pairwise comparisons (*p* value)
Demographic data						
Gender (female/male)	122	31/7	20/23	30/11	**χ**^**2**^ **= 12.4 (*p* = 0.002)**	**CSP ≠ CPP (*p* = 0.002)** **CSP ≠ HC (*p* = 0.033)**
Age (years)	122	50.9 (12.2)	51.8 (12.2)	52.5 (10.4)	K **=** 0.2 (*p* = 0.836)	-
Education	120	15.6 (4.7)	13.2 (4.7)	14.5 (3.3)	**K = 3.1 (*p* = 0.048)**	HC – CPP (*p* = 0.452) HC – CSP (*p* = 0.330) CSP < CPP (*p* = 0.067)
Pain-related clinical data						
Pain intensity (NRS)	109	5.00 (2.4)	6.8 (1.9)	0.90 (1.8)	**K = 83.4 (*p* < 0.001)**	**HC < CPP (*p* < 0.001)** **HC < CSP (*p* < 0.001)** **CPP < CSP (*p* = 0.004)**
Illness duration (months)	79	100.8 (103.9)	74.3 (60.2)	-	χ^**2**^ **=** 0.721 (*p* = 0.392)	-

Significant differences between groups are in bold. CSP, Chronic Secondary Pain; CPP, Chronic Primary Pain; HC, healthy controls; NRS, Numeric Rating Scale.

The sample size (at least 114 participants) was estimated using G-Power software version 3.1 (Faul et al., 2007). Based on previous studies, we expected a small effect size (*f* = 0.20) considering between-group difference in emotion recognition performance and ran *a-priori* within-between interaction repeated measures ANOVA (*α* = 0.05, power = 0.95, number of groups = 3, number of measurements = 6). As repeated measures ANOVA test typically violates the assumptions of sphericity, we established a conservative nonsphericity correction using the formula 1/1 − *m*, where m represents the number of measurements (see Hobson and Bishop, 2016 – Appendix A).

### 2.3 Cognitive and psychopathological assessment

All patients underwent a cognitive and behavioral examination, which started with the self-reporting of the current perceived pain intensity, rated through the Numeric Rating Scale (NRS) (e.g., Williamson and Hoggart, 2005) with a score ranging from 0 (no pain at all) to 10 (the worst imaginable pain).

The following standardized tests were used to measure cognitive and social cognition abilities:

- The cognitive reserve was measured using the Cognitive Reserve Index questionnaire (CRIq) (Nucci et al., 2012), which consists of three indicators addressing: (i) education attainment, i.e., the number of completed years of formal education (school and training course); (ii) occupation attainment, referring to professional position/activity, measured on the intellectual involvement degree and personal responsibility, ranging from 1 = low skilled manual work to 5 = highly responsible or intellectual occupation; (iii) leisure time attainment, namely activities in which individuals engage in their spare time, including intellectual, social and physical activities (measured in frequency and number of years each activity was carried out). The total score is calculated by summing the values associated with the three indicators, with higher scores corresponding to higher levels of cognitive reserve.
- The global cognitive functioning was measured using the Montreal Cognitive Assessment (MoCA) (Nasreddine et al., 2005; Santangelo et al., 2015), which consists of five sub-scores assessing visuo-spatial, language, attention, executive functions, and orientation abilities. The total score is obtained by summing the values associated with the sub-scores, with higher scores indicating a better cognitive functioning.
- The Story-Based Empathy Task (SET) (Dodich et al., 2015) was used to assess individuals’ theory-of-mind functions, namely the ability of attributing mental states to others. It comprises three subscales evaluating individuals’ ability to infer others’ intentions (intention attribution – SET-IA) and emotions (emotion attribution – SET-EA), and individual ability to infer physical causality, which can be considered a control condition (causal inferences – SET-CI). Each subscale consists of six vignettes, requiring selecting the correct ending of a comic strip, which has an upper row containing the story, and a lower row with three options representing the possible conclusions. The task was run on a computer with participants seated at 50 cm distance from the screen (14 inches, 31×17 cm). They were required to select the correct ending, that gives 1 score point. After participants’ response, the following strip was presented. Each subscale has a maximum score of 6 points, and the global score is computed by summing the correct answers, up to a maximum of 18 points, with higher scores indicating a better performance.
- The Ekman-60 faces test (Ekman-60F) (Young et al., 2002; Dodich et al., 2014) consists of a computerized 60 black-and-white pictures of 4 males and 6 females, each displaying the six basic emotions: anger, surprise, fear, disgust, happiness, and sadness. Each stimulus was presented at the center of the screen for 5 s, after that participants choose the correct answer picking from the six basic emotions labels appearing at the bottom of the screen. After participants’ response, the following face was presented. The order of trials was fixed for all subjects. Participants’ accuracy was recorded.

The psychopathological evaluation included the following self-administered scales:

- The Hospital Anxiety and Depression Scale (HADS), that comprises two 7-items subscales assessing anxiety (HADS-A) and depressive (HADS-D) symptoms in nonpsychiatric outpatients with physical illness (Zigmond and Snaith, 1983; Costantini et al., 1999). Higher scores indicate higher anxiety and depressive symptoms.
- The revised version of the Coping Strategies Questionnaire (CSQ-R-I). which assesses the strategies most used by participants to face pain conditions, namely Catastrophizing (6 items) and Praying (3 items), which constitute the “Maladaptive” coping strategies, and Distance (4 items), Ignoring Pain (5 items), Self-Affirmation (4 items) and Distraction (5 items), composing the “Adaptive” coping strategies. The scale includes 27 items evaluated on a Likert scale ranging from 0 = never used to 6 = always used (Riley and Robinson, 1997; Monticone et al., 2014), with higher scores suggesting an extensive use of the coping strategies category.
- The Pain Catastrophizing Scale (PCS), that investigates catastrophism and its subcomponents: rumination (5 items), magnification (2 items), and helplessness (6 items), comprising 13 items evaluated on a Likert scale ranging from 0 = never to 5 = always used (Sullivan et al., 1995; Monticone et al., 2012b), with higher scores indicating higher levels of catastrophism.
- The Fear-Avoidance Behavioral Questionnaire (FABQ), that includes two subscales investigating the pain-specific behavior of avoiding work (FABQ_W, 11 items) and physical activity (FABQ_PA, 5 items). All the items were evaluated on a Likert scale ranging from 0 = completely disagreed to 6 = completely agreed (Waddell et al., 1993; Monticone et al., 2012a). Higher total scores indicated more extensive fear-avoidance behaviors.
- The Eurohis-QoL 8-item Index, which assesses the overall quality of life. It includes eight items evaluated on a Likert scale ranging from 0 = completely unsatisfied to 4 = completely satisfied (Schmidt et al., 2006; Schiavolin et al., 2015). Higher scores indicate a higher quality of life.
- The Toronto Alexithymia Scale (TAS-20), used for the assessment of alexithymia, namely the inability to recognize or describe own emotions (Bagby et al., 1994; Bressi et al., 1996). Higher scores correspond to higher levels of alexithymia.

### 2.4 Procedure

Two experienced neuropsychologists (A.T. and M.C.) administered the cognitive and behavioral assessment. During the clinical interview, demographical information and experienced pain intensity were collected. After that, the cognitive assessment was performed administering CRIq, MoCA, SET and Ekman-60F tests. Finally, participants were asked complete the psychopathological questionnaires. The protocol was administered in one single session of one hour and a half and the order of cognitive and behavioral tests was fixed as described.

### 2.5 Statistical approach

We performed analyses in the statistical programming environment R (R Core Team, 2023).

Considering the Ekman-60F task, the dichotomous variable accuracy was analyzed using general mixed effects models (Baayen, 2012), fitted using the GLMER function of the lme4 R package (Bates et al., 2015). *Group* (factorial, three levels: CPP, CSP, HC), *Emotion* (factorial, six levels: anger, disgust, fear, happiness, sadness, and surprise), and their interaction were entered in the full model as fixed factors. Moreover, we added the simple effect of *MoCA* total score to account for the effect of individuals’ global cognitive efficiency of participants on task performance. By-subject and by-item random intercepts were included to account for participant-specific variability and item-specific idiosyncrasies (Baayen, 2012). The inclusion of fixed predictors in the final model has been tested with a series of likelihood ratio tests by progressively removing parameters that did not significantly increase the overall model goodness of fit (Gelman and Hill, 2006) (see Supplementary Table S1 for details on the best fitting model selection).

Concerning SET performance, residuals normality was plotted using the olsrr package (Hebbali and Hebbali, 2017). Since residuals were normally distributed in SET total scores and subscales, we ran linear model analyses with the function lm of lme4 package including Group and MoCA scores as fixed factors on four independent models including the SET total scores and the three subscales (SET – IA, SET – EA, SET – CI) as dependent variables.

Post-hoc analyses of Ekman-60F and SET tasks were performed using the testInteractions function of the phia package (De Rosario-Martinez et al., 2015).

Exploratory correlations were run separately for each group, to investigate specific relationships among cognitive and psychopathological variables. Partial correlations were run using the partial.r function implemented in the psych package, controlling for age and education (Revelle, 2022). Pearson-correlation coefficients and two tailed probabilities applying Bonferroni correction were computed. The correlation matrix was plotted using the corrplot package (Wei et al., 2017) (see also Petilli et al., 2021 for available R script).

The dataset and script used for the analyses have been released on a public data repository: 10.5281/zenodo.10562236.

## 3 Results

### 3.1 Cognitive and pain-related clinical assessment

Participants’ scores on cognitive tests and psychopathological questionnaires, as well as statistical differences among groups are reported in Tables 2, 3.

**Table 2:** Cognitive performance in CSP, CPP, and HC.

	No.	CPP Mean (SD)	CSP Mean (SD)	HC Mean (SD)	Coefficients of Fisher test (*p* value)	Pairwise comparisons (*p* value)
Cognitive reserve (CRIq)	119	115 (15.3)	108.3 (16.1)	117.7 (14.4)	**4.0 (*p* = 0.022)**	HC – CPP (*p* = 0.693) **CSP < HC (*p* = 0.017)** CSP – CPP (*p* = 0.150)
CRIq – Education	119	109.9 (15.6)	103.2 (14.9)	108.4 (11.9)	2.21 (*p* = 0.117)	-
CRIq – Work	119	103.3 (12.2)	105.5 (14.7)	112.2 (15.12)	**4.3 (*p* = 0.017)**	**HC > CPP (*p* = 0.014)** HC – CSP (*p* = 0.112) CSP – CPP (*p* = 0.744)
CRIq – Leisure activities	119	120.6 (16.5)	109.9 (16.5)	119.5 (14.9)	**5.2 (*p* = 0.008)**	HC – CPP (*p* = 0.949) **CSP < HC (*p* = 0.020)** **CSP < CPP (*p* = 0.015)**
Global cognitive functioning (MoCA)	120	24.6 (3.2)	23.7 (2.8)	25.5 (2.4)	**5.19 (*p* = 0.008)**	HC – CPP (*p* = 0.317) **CSP < HC (*p* = 0.005)** CSP – CPP (*p* = 0.361)

Significant between groups differences are in bold. CSP, Chronic Secondary Pain; CPP, Chronic Primary Pain; HC, healthy controls; NRS, Numeric Rating Scale; CRIq, Cognitive Reserve Index – Questionnaire; MoCA, Montreal Cognitive Assessment: raw scores were used for analysis; participants with equivalent score = 0 were excluded from analysis (Santangelo et al., 2015).

**Table 3:** Psychopathological evaluation of CSP, CPP, and HC.

	No.	CPP Mean (SD)	CSP Mean (SD)	HC Mean (SD)	Coefficients of Fisher test (*p* value)	Pairwise comparisons (Sign)
Anxiety (HADS-A)	118	9.02 (4.9)	7.7 (5.1)	5.4 (3.5)	**7.6 (*p* = 0.001)**	**HC < CPP (*p* < 0.001)** HC – CSP (*p* = 0.055) CSP – CPP (*p* = 0.482)
Depression (HADS-D)	118	6.00 (4.4)	6.2 (4.3)	3.4 (2.9)	**7.8 (*p* < 0.001)**	**HC < CPP (*p* = 0.010)** **HC < CPS (*p* = 0.003)** CSP – CPP (*p* = 0.983)
Adaptive coping (CSQ-I-R)	117	0.5 (0.2)	0.5 (0.2)	0.5 (0.2)	0.43 (*p* = 0.650)	-
Maladaptive coping (CSQ-I-R)	117	0.4 (0.3)	0.5 (0.3)	0.2 (0.2)	**16.2 (*p* < 0.001)**	**HC < CPP (*p* = 0.001)** **HC < CSP (*p* < 0.001)** CSP – CPP (*p* = 0.856)
Helplessness (PCS)	116	0.6 (0.5)	0.5 (0.2)	0.1 (0.1)	**32.8 (*p* < 0.001)**	**HC < CPP (*p* < 0.001)** **HC < CSP (*p* < 0.001)** CSP – CPP (*p* = 0.487)
Rumination (PCS)	116	0.7 (0.5)	0.6 (0.3)	0.3 (0.2)	**12.4 (*p* < 0.001)**	**HC < CPP (*p* = 0.002)** **HC < CSP (*p* < 0.001)** CSP – CPP (*p* = 0.468)
Magnification (PCS)	116	0.4 (0.3)	0.3 (0.3)	0.3 (0.2)	2.4 (*p* = 0.094)	-
Quality of Life (EuroHis-QoL)	119	2.3 (0.7)	2.2 (0.8)	3.6 (0.6)	**54.2 (*p* < 0.001)**	**HC > CPP (*p* < 0.001)** **HC > CSP (*p* < 0.001)** CSP – CPP (*p* = 0.580)
Alexithymia (TAS-20)	117	51.3 (10.9)	43.5 (14.8)	42.6 (9.5)	**7.2 (*p* = 0.001)**	**HC < CPP (*p* < 0.001)** HC – CSP (*p* = 0.196) CSP – CPP (*p* = 0.417)
Physical activity avoidance (FABQ-pa)	115	11.5 (8.2)	15.7 (9.2)	6.3 (5.9)	**15.4 (*p* < 0.001)**	**HC < CPP (*p* = 0.009)** **HC < CSP (*p* < 0.001)** CSP – CPP (*p* = 0.098)
Work activity avoidance (FABQ-w)	113	11.9 (12.5)	11.7 (13.5)	7.8 (9.7)	1.7 (*p* = 0.185)	-

Significant between groups differences are in bold. CSP, Chronic Secondary Pain; CPP, Chronic Primary Pain; HC, healthy controls; NRS, Numeric Rating Scale; HADS-A, Hospital Anxiety Depression Scale – Anxiety; HADS-D, Hospital Anxiety Depression Scale – Depression; CSQ-I-R, Coping Strategies Questionnaire – Italian – Revised; PCS, Pain Catastrophism Scale; EuroHis-QoL, EuroHis Quality of Life scale; TAS-20, Toronto Alexithymia Scale 20 items; FABQ – pa, Fear-Avoidance Belief Questionnaire – Physical Activity; FABQ – w, Fear-Avoidance Belief Questionnaire – work.

### 3.2 Ekman-60F results

First, we performed a preliminary check of accuracy in the three groups of participants (see Supplementary Figure S2). One participant in the CPP group had a low level of accuracy (55%) compared to the other participants in the same group and was removed from the analyses. Therefore, the analyzed sample included 121 participants and statistical analyses were run on 7,260 data points. The best-fitting model included the simple effects of Emotion [χ^2^_(5)_ = 70.8, *p* < 0.001], Group [χ^2^_(2)_ = 17.9, *p* < 0.001], and MoCA [χ^2^_(1)_ = 19.3, *p* < 0.001].

Considering the simple effect of emotion, post-hoc analyses with false discovery rate correction (FDR) highlighted that happiness was recognized more accurately than all the other emotional categories (all *p*s < 0.002), followed by surprise that was better recognized compared to the other four emotions (*p*s < 0.042). The most difficult emotion to be recognized was fear (all *p*s < 0.021). Table 4 summarizes the means (percentage) and standard deviations of performance in the six basic emotions.

**Table 4:** Mean (percentage) and standard deviations of accuracy performance in the six basic emotions, calculated on the overall sample.

Anger	Disgust	Fear	Happiness	Sadness	Surprise
71.6 ± 46.1	79.3 ± 40.5	54.5 ± 49.8	98.3 ± 12.8	78.9 ± 40.8	91.2 ± 28.3

**Figure 1:**
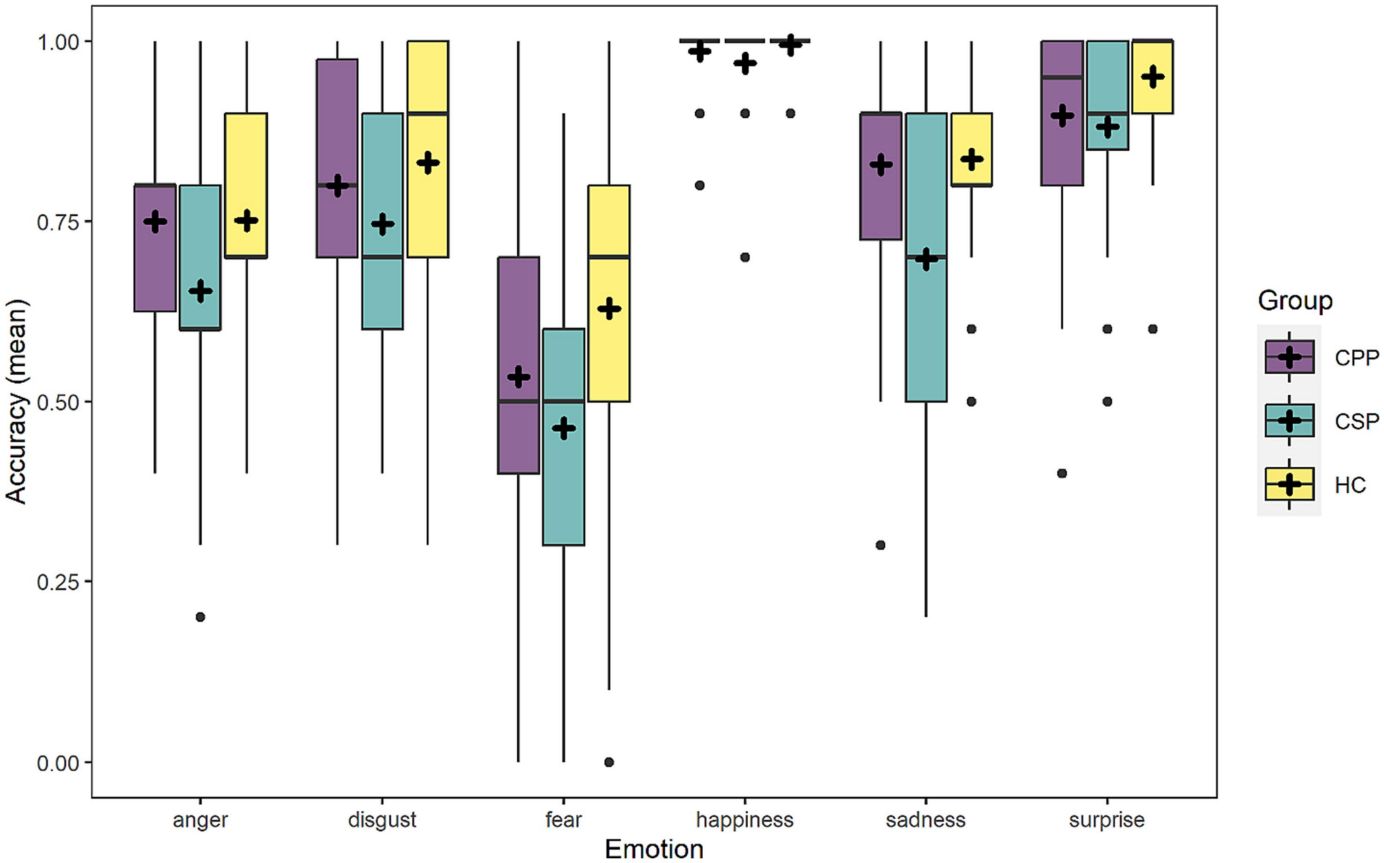
The figure graphically represents the accuracy of the three groups in the six emotions depicted in the Ekman-60F. Dots represent outliers.

As for the simple effect of group, CSP participants (mean = 73.5, SD ± 44.1) were less accurate than HC and CPP (*p* < 0.001 and *p* = 0.003, respectively), while HC (mean = 83.3, SD ± 37.3) and CPP (mean = 80.6, SD ± 39.5) performance did not significantly differ (*p* = 0.346). Figure 1 represents performance distribution in the Ekman-60F task. Lastly, the simple effect of MoCA revealed lower emotion recognition accuracy at lower levels of global cognitive efficiency.

### 3.3 Set results

One participant from the CPP group was removed because she did not perform the task. Mirroring the preliminary procedure of Ekman-60F analyses, we performed a preliminary check of SET total scores performance in the three groups (see Supplementary Figure S3 for outliers’ visualization). Four participants with SET total scores lower than 12 in HC, and two participants with scores lower that 7 from the CPP were removed from subsequent analyses, which were run on 115 participants.

**Figure 2:**
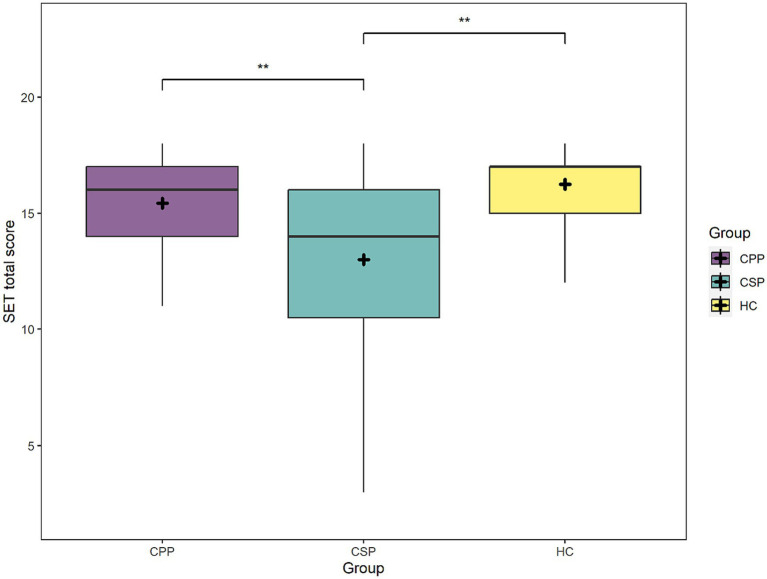
The figure depicts mean scores at the SET task (total scores) of each group. Asterisks represent statistical *p*-values ** *p* < 0.01.

Considering the SET global scores, the factors Group [*F*_(2,109)_ = 7.9, *p* < 0.001] and MoCA scores [*F*_(1,109)_ = 33.2, *p* < 0.001] significantly predicted participants performance. CSP performed worse than HC and CPP (*p* = 0.001 and *p* = 0.003, respectively), while no difference was found between HC and CPP (*p* = 0.577) (see Figure 2). As expected, performance at SET decreased at lower cognitive efficiency as measured by MoCA.

As for the intention attribution (SET-IA) scores, the effect MoCA was significant [*F*_(2,109)_ = 13.6, *p* < 0.001] in predicting performance, with better performance at the SET subscale at higher MoCA scores. Conversely, the effect of Group showed a trend that did not reach significance [*F*_(2,109)_ = 2.7, *p* = 0.069].

**Figure 3:**
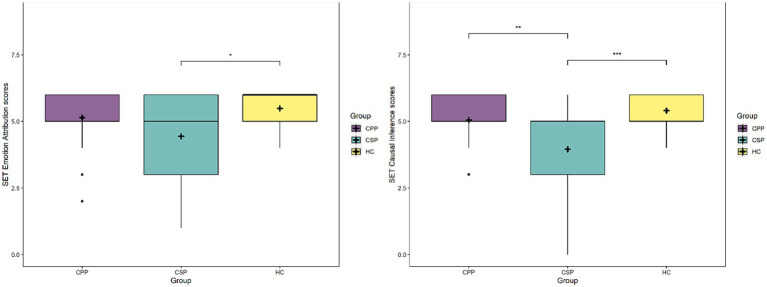
The figure depicts mean scores at the SET-EA subscale (left panel) and SET-CI (right panel) for each group. Asterisks represent statistical *p*-values *** *p* < 0.001, ** *p* < 0.01, * *p* < 0.05.

Considering the emotion attribution subscale (SET-EA), Group [*F*_(2,109)_ = 3.6, *p* = 0.030] and MoCA scores [*F*_(2,109)_ = 24.6, *p* < 0.001] significantly predicted performance, with CSP performing significantly worse than HC (*p* = 0.032), while CPP did not differ from HC (*p* = 0.469) and showed a trend compared to CSP (*p* = 0.085) (see Figure 3 - left panel). Again, lower MOCA scores corresponded to a lower performance at SET-EA.

Lastly, considering the causal inference subscale (SET-CI), Group [*F*_(2,109)_ = 8.8, *p* < 0.001] and MoCA scores [*F*_(2,109)_ = 23, *p* < 0.001] significantly predicted performance: once again CSP performed worse than HC and CPP (*p* < 0.001 and *p* = 0.002, respectively), while the latter did not differ (*p* = 0.530) (see Figure 3 - right panel). Performance at SET increased at higher MoCA scores.

### 3.4 Exploratory results from correlations including cognitive, psychopathological, and pain-related clinical variables

In the CPP group, we did not find correlations between variables related to pain and other cognitive or psychopathological variables (see Supplementary Figure S4). Significant correlations appeared only between psychopathological variables (e.g., anxiety – depression, helplessness – rumination – magnification). In the CSP group, correlations between psychopathological variables were found similarly to CPP group, but even in this case, no correlations emerged between pain-related and cognitive or psychopathological variables (Supplementary Figure S5). As highlighted by the regression models, only in CSP, global cognitive functioning (i.e., MoCA) scores were positively correlated with performance in emotion recognition and theory of mind (*r* = 0.61, *p* = 0.002 and *r* = 0.55, *p* = 0.015, respectively). See Supplementary materials, section C for further details on exploratory correlation results.

## 4 Discussion

CPP and CSP diagnoses have been recently implemented in the ICD-11 to distinguish between chronic pain as the primary cause of the patient’s complaint (CPP) and chronic pain secondary to other conditions (CSP). Since the novelty of the classification, no previous evidence is available considering possible similarities and differences in the cognitive profile of the two conditions.

In the present work, we aimed at contributing to the characterization of the two disorders by assessing social cognitive abilities in CPP and CSP patients compared to an age-matched healthy control group. We administered two validated tasks, namely the Ekman-60F, that assesses facial emotion recognition, and the SET, that investigates the individuals’ theory of mind or empathy functions, namely the ability of attributing mental and emotional states to others. Moreover, we compared CPP and CSP considering demographical, clinical pain-related and psychopathological dimensions which were previously considered by two retrospective studies (Hornemann et al., 2020; Munk et al., 2022).

In line with Hornemann et al. (2020), we found a larger prevalence of women among CPP than CSP. Differently from this study, we did not find longer pain duration, and the pain intensity measured at the time of the assessment was higher for CSP patients than CPP. CSP patients reached lower scores than CPP in the cognitive reserve subscale considering leisure activities. We did not find differences between CPP and CSP in coping strategies and catastrophism, similarly to Munk et al. (2022), and neither for mood and anxiety symptoms, and quality of life, in line with Hornemann et al. (2020).

Considering facial expressions recognition, we found that CPP and CSP differed in the ability to identify emotions. Crucially, only CSP patients were impaired with respect to healthy controls, while CPP did not statistically differ from the control group. The result is in contrast with previous findings on patients with fibromyalgia (Weiß et al., 2013; Guevara et al., 2021), low back pain (Grabli et al., 2022) and other chronic pain disorders included in the new CPP category, where an impairment in emotion recognition was found compared with healthy participants. Our results suggest indeed that only patients in which pain was a consequence of another medical condition showed a clinically significant impairment in emotion recognition. In line with the previous literature considering chronic pain (Shin et al., 2013; Sohn et al., 2016), no interactions emerged between participants’ group and emotions, suggesting an overall impairment in recognizing facial expressions. A simple effect of emotions emerged independently from the group, with happiness being the easiest emotion to recognize, followed by surprise. Fear, instead, was the most difficult emotion to identify, correctly detected only in the 55% of trials. This pattern confirmed previous evidence (Mattavelli et al., 2019; Vergallito et al., 2020; Jiskoot et al., 2021; Mattavelli et al., 2021) showing that some emotions are easier to detect compared to others (Young et al., 2002; Dodich et al., 2014), probably due to the presence of highly salient and distinctive facial features.

Considering the theory of mind abilities, as assessed by SET global scores, CSP resulted more compromised, whereas CPP were not impaired compared to HC. At a closer look, although CSP had lower scores in all the three SET subscales, such difference reached significance in comparison with CPP only in the causal inference subscale and nearly in the emotion attribution subscale. Compared to healthy controls, instead, CSP were impaired in both subscales but not in the intention attribution task. Since the aim of this work was tracing a profile for CPP and CSP diagnoses, results at SET should be interpreted with caution. Indeed, lower performance in the SET causal inference subscale – which is considered a control condition – may suggest the presence of impairment in general executive functions, rather than specific dysfunction in the theory-of-mind ability (Dodich et al., 2015).

Our analyses highlighted that the global cognitive functioning also impacted emotion recognition and empathy tasks. Indeed, individuals were more accurate in recognizing emotions and attributing intentions and emotions at higher MoCA scores. This effect can be considered in line with previous studies (Guevara et al., 2021; Raimo et al., 2022) suggesting that patients affected by fibromyalgia (Guevara et al., 2021) and chronic migraine (Raimo et al., 2022) failed in both social cognition and executive functioning tasks, whose performance was positively correlated.

Converging evidence suggested that impairment in cognition can be considered a comorbidity with chronic pain (Wiech et al., 2008; Moriarty et al., 2011), although the mechanisms underlying such relationship are far from being understood (Phelps et al., 2021). Some authors explained this phenomenon by considering that individuals have limited cognitive resources, and the presence of stimuli that prioritize attention (likewise pain) occupies a significant proportion of such resources to the detriment of other tasks (Eccleston, 1994). This partially aligns with our results: indeed, we found that individuals with CPP and CSP engage more frequently than healthy controls in catastrophizing thoughts, rumination, and helplessness feelings (Table 3). Such recursive processes require considerable attention such as keeping the individuals monitoring any pain signal from their body (Legrain et al., 2009) and reducing threshold intrusion of the expected information (i.e., pain) at the conscious level (Wells and Matthews, 1996). Although we found that these psychopathological measures correlated to each other, we did not find correlations with the cognitive measures, namely the MoCA scores, or social cognition abilities.

Significant differences between CSP and HC were found in cognitive reserve, were CSP has lower scores than HC and CPP especially in the leisure activities section, that is the most related to the social and relational aspects of human life (Nucci et al., 2012). Finally, in both patients’ group, the illness duration and pain intensity did not correlate with other psychopathological and/or cognitive measures.

Taken together our findings support the importance of examining the domain of social cognition in chronic pain, and especially in secondary chronic pain conditions. As previously discussed, indeed, the existing literature focused on pathologies which are now comprised in the CPP category, suggesting an impairment in social cognition in patients compared to controls. Differently from literature, our study suggests that impairment in social cognition is greater in CSP than CPP, while no differences emerged between CPP and HC. Considering this latter point, it seems possible to hypothesize that deficits in social cognition are not linked to chronic pain *per se* and pave the way to future studies that should clarify whether impairment in social cognition could be related to the primary cause of pain or maybe represent a *vulnerability* to the emergence and persistence of pain symptoms when a primary medical condition occurs. This hypothesis is in line with some experimental studies suggesting that higher cognitive abilities result in a better tolerance to pain induction (Pickering et al., 2002; Zhou et al., 2015). Moreover, previous studies with populations at risk for developing chronic postsurgical pain or cancer pain, suggested that limited cognitive capacity, as well as high psychosocial vulnerabilities, are predictive of the severity and prevalence of pain after the intervention, thus supporting our hypothesis that premorbid individuals’ features may contribute to the emergence and maintenance of pain following a painful event (Thomas et al., 1999; Voute et al., 2020; Rouch et al., 2021). Thus, future studies should clarify whether deficits in social cognition may contribute to such emergence.

Consistently, considering the differences emerged between CSP and HC not only in the emotion recognition task, but also in cognitive reserve and leisure activities, our results highlight the need for greater attention to social cognition and social functioning, considered crucial for the quality of life of social beings like humans (Frith and Frith, 2007). Moreover, the findings that changes in social cognition are dissociable from mood and pain-related coping strategies argue in favor of including social cognition in the diagnostic assessment of chronic pain and potentially in multidisciplinary pain therapy (Cheatle, 2016), suggesting that a comprehensive neuropsychological assessment can help to better understand how psychological and social factors interact with physical symptoms.

As far as we know, this is the first study assessing the social cognition performances in CSP, by comparing it with CPP and healthy volunteers, and certainly more studies are needed to further investigate differences between CPP and CSP cognitive and psychopathological profile. This would help to clarify whether our findings are due to specific features of our sample or can be representative to CPP and CSP patients.

### 4.1 Limitations of the present research

The main limit of our study is gender discrepancies in our sample. CPP group includes more women than men, a difference already acknowledged among pain populations, revealing that primary chronic pain incidence is higher in women (Hornemann et al., 2020). Further studies should balance participants for gender, possibly involving larger samples to explore differences in cognitive functioning and/or impairment (Fillingim, 2017) and social cognition abilities (Gur et al., 2010; Kret and De Gelder, 2012; Christov-Moore et al., 2014). A second limitation is that typically literature on schizophrenic patients considers four different domains when targeting social cognition, namely emotion recognition, theory of mind, social perception, social knowledge and attributional bias (Green et al., 2019). In the present study we only targeted the first two domains, as did the prevalent literature on social cognition (Kurtz et al., 2016; Green et al., 2019). Considering our results as pioneering, future studies should expand the investigation also targeting the other domains of social cognition.

## Data availability statement

The raw data supporting the conclusions of this article have been made available by the authors, without undue reservation.

## Ethics statement

The studies involving humans were approved by Ethical Board Committee of the Fondazione IRCCS Istituto Neurologico “Carlo Besta,” Milano. The studies were conducted in accordance with the local legislation and institutional requirements. The participants provided their written informed consent to participate in this study.

## Author contributions

AT: Conceptualization, Data curation, Methodology, Writing – original draft. AV: Conceptualization, Data curation, Formal analysis, Writing – original draft. MC: Conceptualization, Writing – review & editing. GM: Funding acquisition, Visualization, Writing – review & editing. AF: Data curation, Writing – review & editing. LG: Writing – review & editing. SU: Funding acquisition, Investigation, Writing – review & editing. LR: Conceptualization, Supervision, Writing – review & editing.
