# Supplementary material for: Social cognition abilities in patients with primary and secondary chronic pain
Source: Front Psychol. 2024 Feb 27;15:1315682. doi: 10.3389/fpsyg.2024.1315682 (PMC11002902; doi:10.3389/fpsyg.2024.1315682)
Supplement: Supplementary file 1 [file Data_Sheet_1.PDF]

# Supplementary Materials

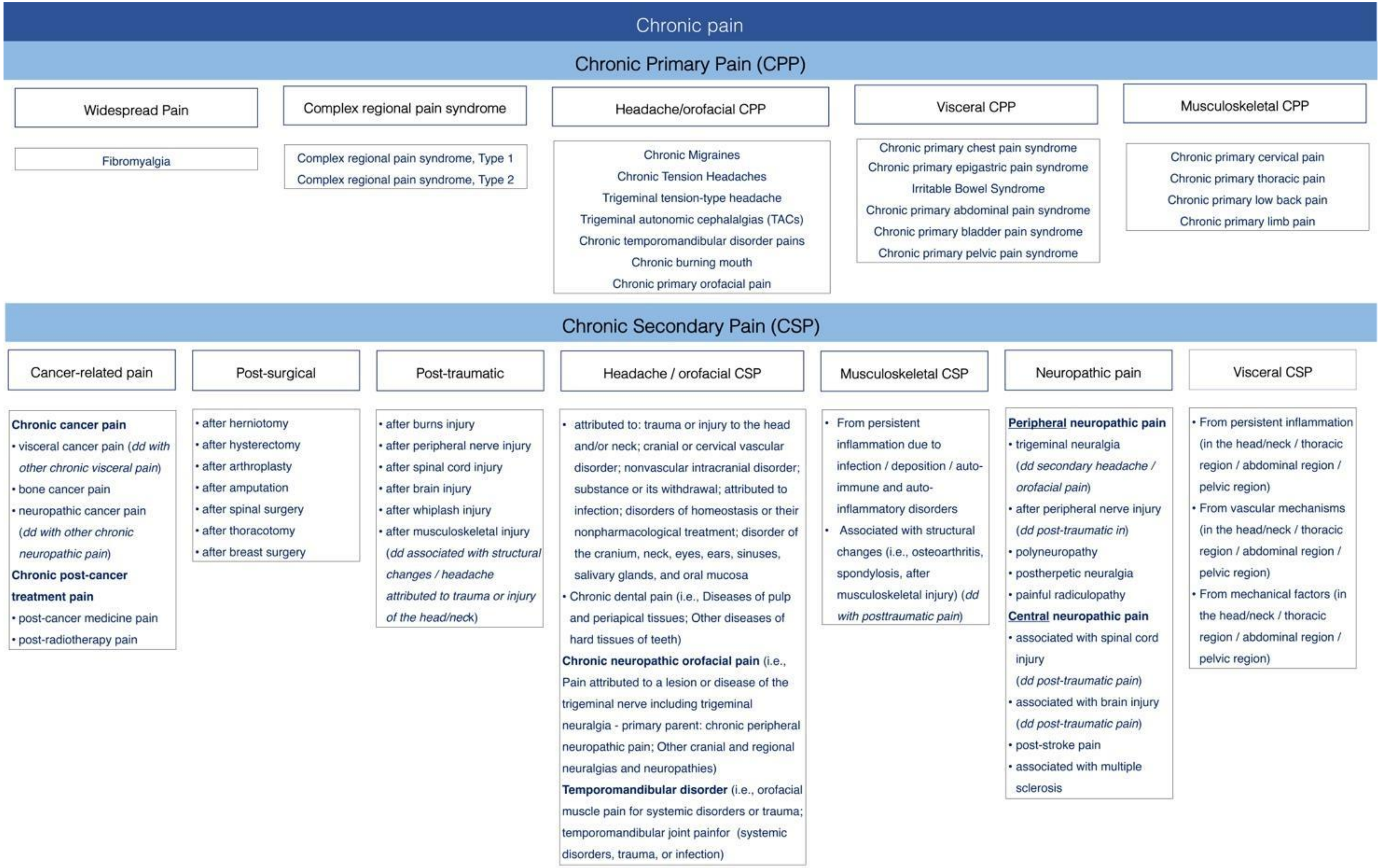


Figure S1 is a graphical scheme of the classification of chronic pain diseases into CPP and CSP, according to the ICD-11 (Aziz et al., 2019; Bennett et al., 2019; Benoliel et al., 2019; Nicholas et al., 2019; Perrot et al., 2019; Scholz et al., 2019; Schug et al., 2019; Treede et al., 2019) .

## Section A: CPP and CSP Categorization

Participants included in the CPP and CSP groups had the following specific diagnoses:

- CPP group: thirty-eight patients included. Seven patients were diagnosed with chronic primary headache, twelve with chronic atypical facial pain, three with fibromyalgia, fifteen with idiopathic small fiber neuropathy, and one with chronic primary pelvic pain.
- CSP group: forty-three patients included. Seven patients were diagnosed with chronic post-traumatic pain, eleven with chronic post-surgical pain, nine with polyneuropathy, five with central neuropathic pain, four with diabetic neuropathy, two with neuropathic cancer pain, one with secondary facial pain, three with auto-inflammatory neuropathic pain, and one with postherpetic neuralgia.

## Section B: Emotion Recognition Task (Ekman-60F)

Figure S2. Outliers' removal in the Emotion Recognition Task

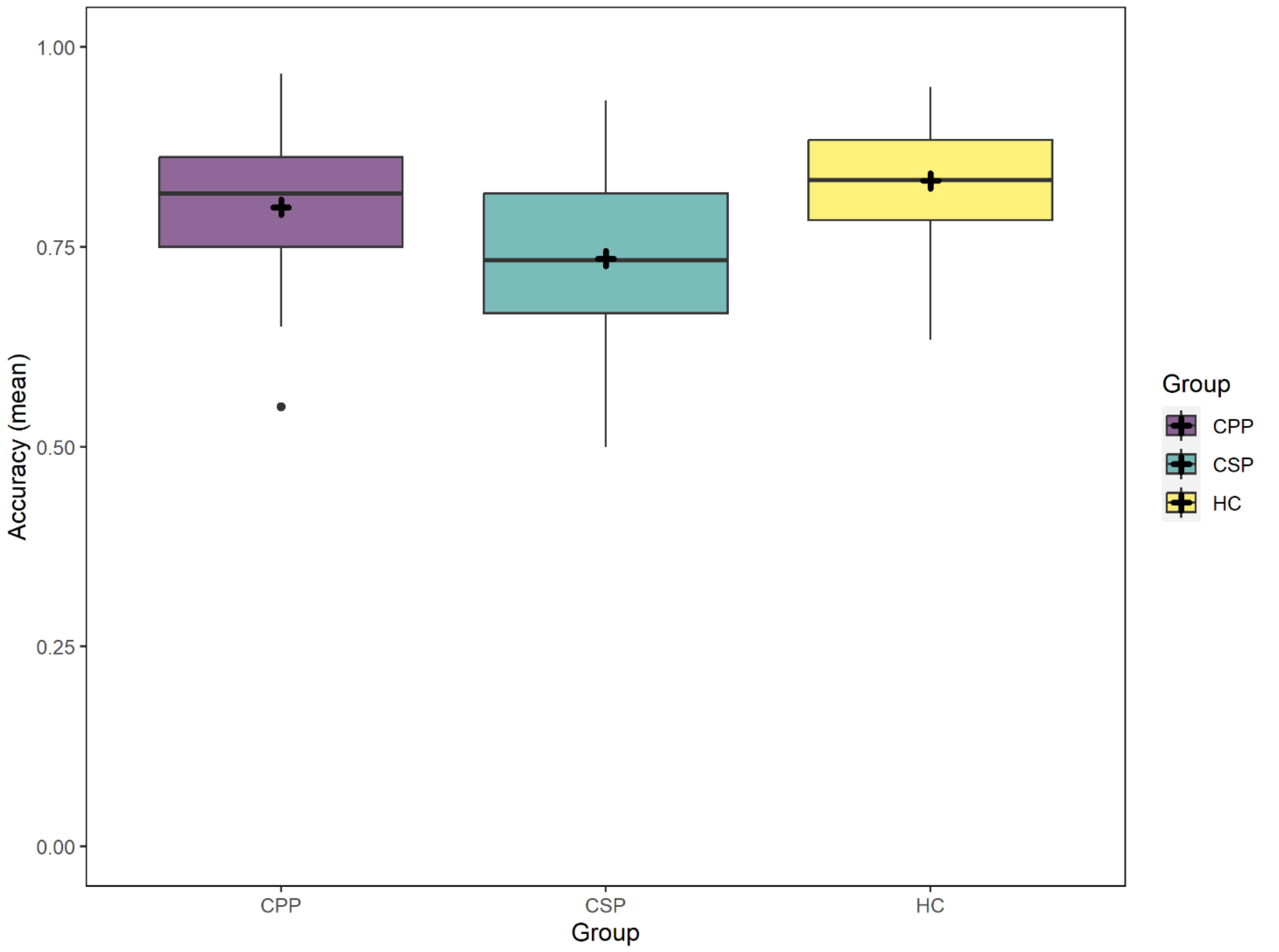


Figure S2 depicts subjects' overall performance in the emotion recognition task according to the assigned group. Only a participant (ID = 59) in the CPP group was classified as an outlier, reaching an accuracy of 55%. Therefore, we removed data points from this participant from the emotion recognition analysis.

***Table S1**. Results of the mixed-effect analysis on Ekman accuracy*

| ***Parameter*** | $\chi^2$ | ***P*** | ***Removal order*** | $\chi^2$ | ***df*** | ***p*** |
|---|---|---|---|---|---|---|
| *MoCA* | - | - | *Not removed* | 19.453 | 1 | <.001 |
| *Group* | - | - | *Not removed* | 17.907 | 2 | <.001 |
| *Emotion* | - | - | *Not removed* | 71.157 | 5 | <.001 |
| *Group * Emotion* | 15.61 | .1114 | 1 | - | - | - |

*Table S1 summarizes the model-simplification procedure, including the goodness-of-fit tests and their results. The rightmost part of each table reports the effects of the included variables.*

Figure S3. Outliers' removal in the Story-Based Empathy Task (SET)

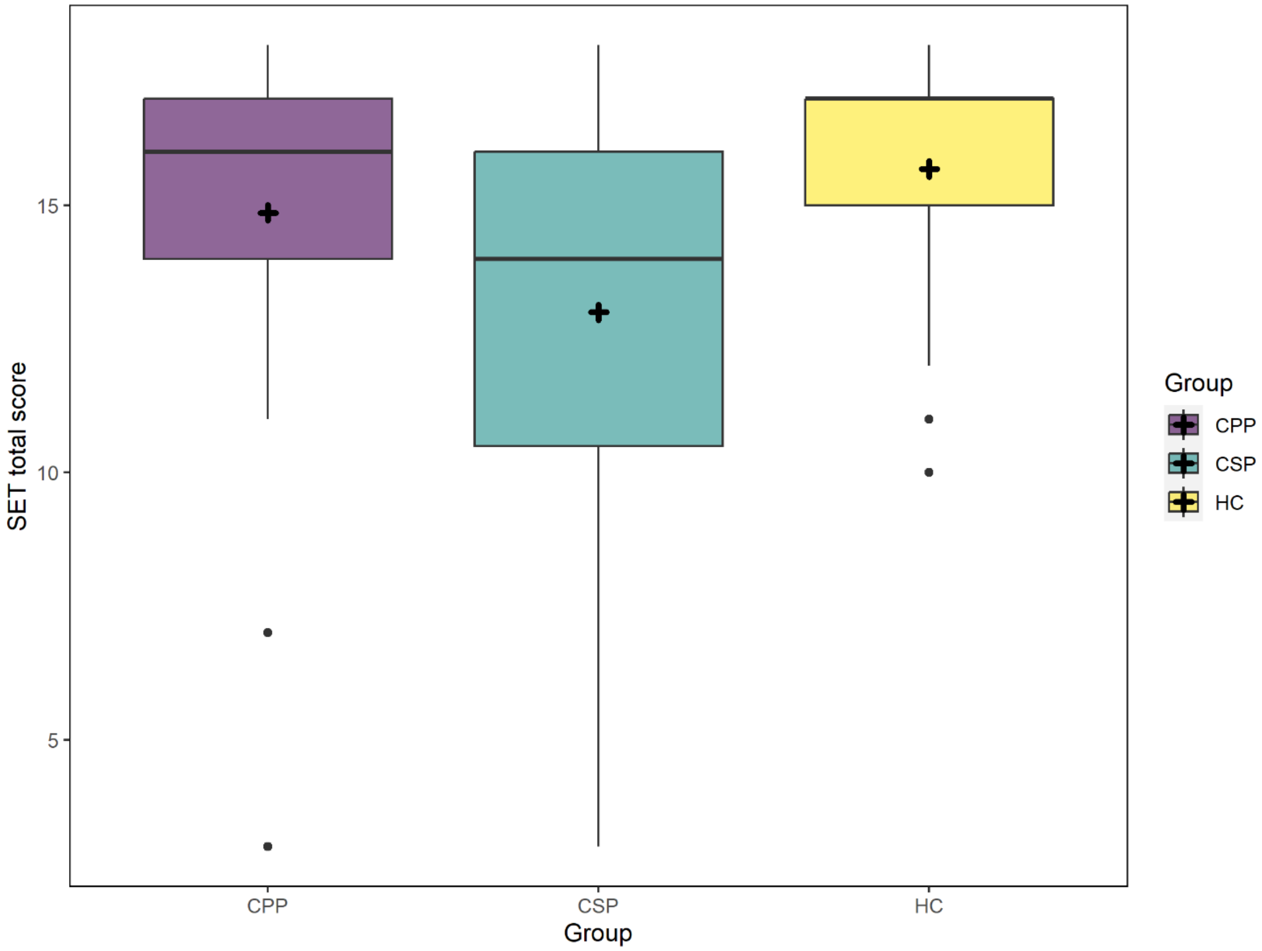


Figure S3 depicts subjects' overall performance in the social cognition task according to the assigned group. Participants with SET total scores lower than 12 in HC (ID = 505, 508, 509, 521) and 7 among CPP (ID = 6, 128) were removed from subsequent analyses.

## Section C: Explorative Correlation Analysis

### Partial correlations between cognitive, psychopathological, and pain-related clinical variables in the CPP sample

We did not find correlations between pain-related variables and other cognitive or psychological variables in the CPP group. Within the group, anxiety and depression scores were positively correlated ($r = 0.71$, $p < .001$), the use of maladaptive coping strategies correlated with anxiety ($r = 0.60$, $p = .009$), helplessness, rumination, and magnification were correlated (all $r > 0.73$, all $p < .001$), alexithymia correlated with depression ($r = 0.62$, $p = .004$). Quality of life was negatively associated with depression ($r = -0.65$, $p = .001$) (see Figure S4).

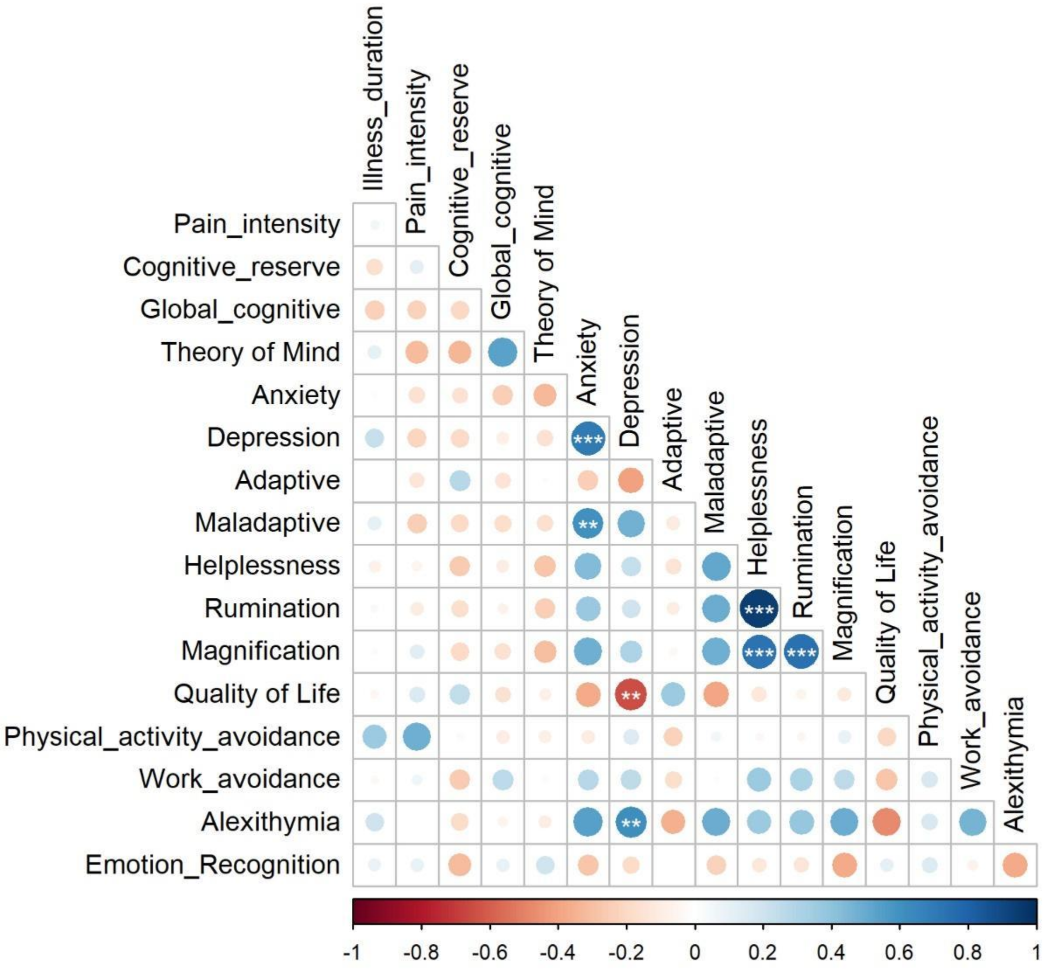


**Figure S4** shows the CPP correlogram or correlation matrix depicting the relationships between each pair of variables. Positive correlations are blue-colored, and negative correlations are red-colored. Dots' color intensity and size are proportional to the correlation coefficients, and asterisks inside the dots represent the statistical significance (* $p < .05$, ** $p < .01$, *** $p < .001$).

**Partial correlations between cognitive, psychopathological, and pain-related clinical variables in the CSP sample**

In the CSP group, some correlations were similar to those found in the CPP group. Even in this case, no correlations emerged between pain-related and cognitive or psychological variables; anxiety and depression were positively correlated (r = 0.77, p < .001). Quality of life was negatively correlated with depression (r = -0.55, p =.021) and positively with adaptive coping strategies (r = 0.52, p= .048). Other correlations were instead specific to this group; for example, the quality of life negatively correlated with the anxiety scores (r = -0.54, p = .024). Magnification correlated with anxiety, depression and alexithymia scores (r = 0.70, p < .001, r =0.59, p =.004, and r =0.58, p = .006, respectively). Moreover, magnification, helplessness, and rumination correlated with maladaptive coping strategies (all r > .57, all p <.010). Considering the social cognition measures, as highlighted by the regression models, MoCA scores were positively correlated with performance in emotion recognition and theory of mind (r = 0.61, p = .002 and r = 0.55, p = .015, respectively) (figure S5).

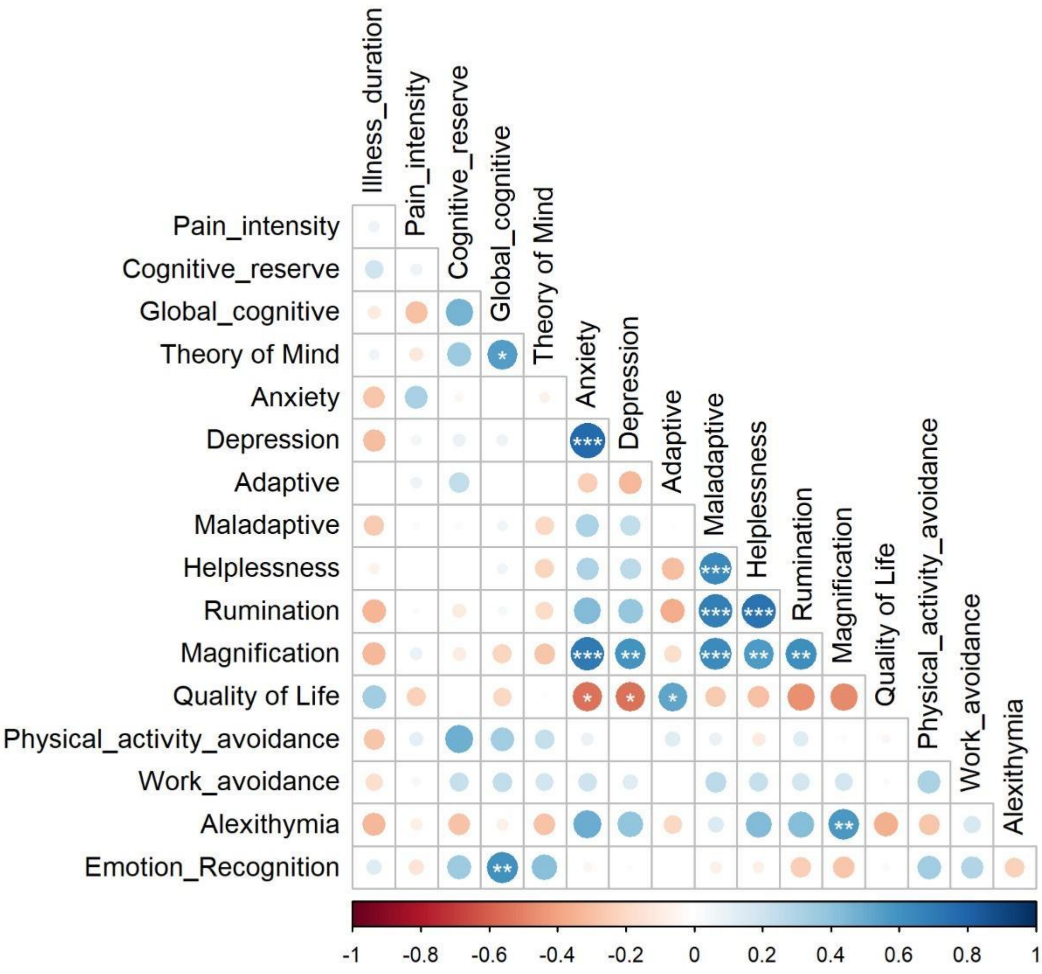


**Figure S5** shows the CSP correlogram or correlation matrix depicting the relationships between each pair of variables. Positive correlations are blue-colored, and negative correlations are red-colored. Dots' color intensity and size are proportional to the correlation coefficients, and asterisks inside the dots represent the statistical significance (* p < .05, ** p < .01, *** p < .001).

**Partial correlations between cognitive, psychopathological, and pain-related clinical variables in the HC sample.**

Considering the healthy control group, positive correlations were found between anxiety and depressive scores (r = 0.59, p = .006), helplessness and depression (r = 0.59, p = .005), rumination with helplessness, maladaptive coping strategies and magnification (all r > 0.61, all p < .002) (Figure S6).

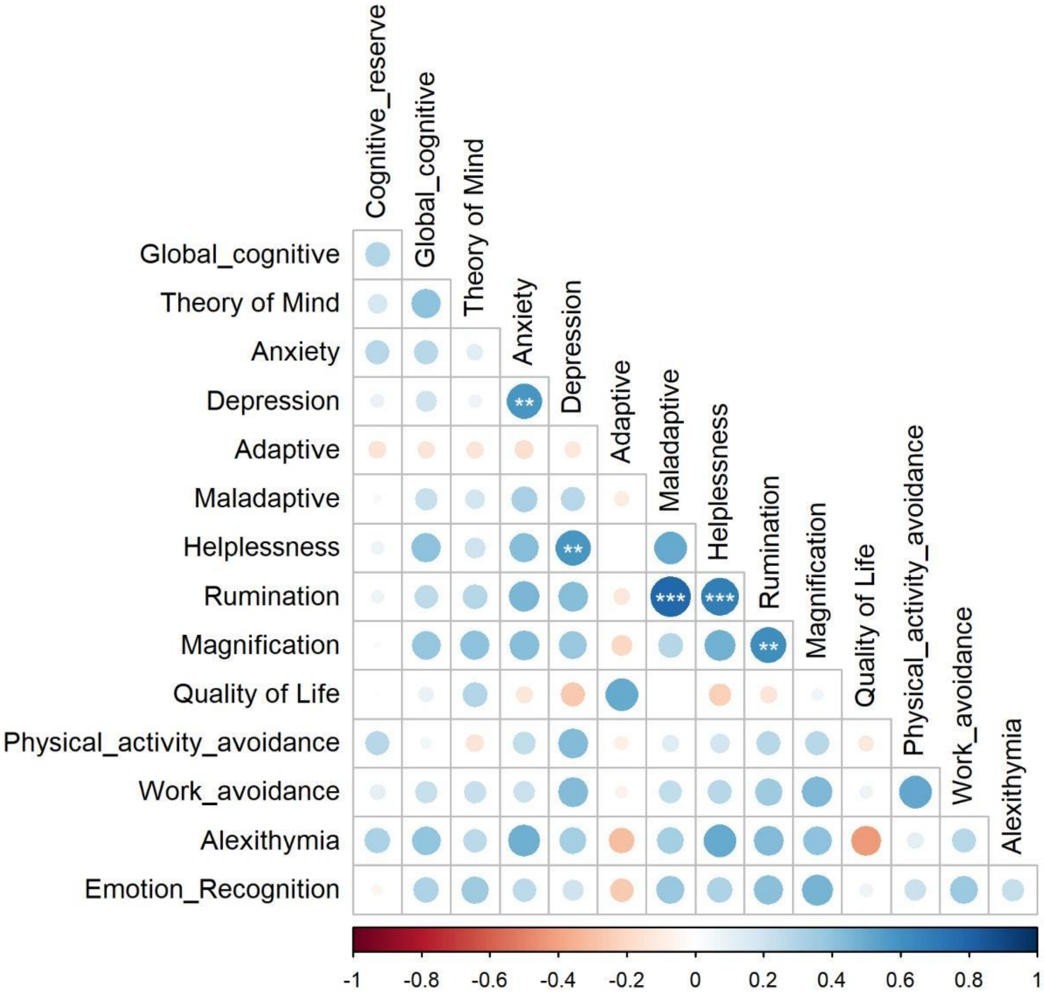


**Figure S6** shows the HC correlogram o correlation matrix depicting the relationships between each pair of variables. Positive correlations are blue colored and negative correlations are red colored. Dots color intensity and size are proportional to the correlation coefficients and asterisks inside the dots represent the statistical significance (* p < .05, ** p < .01, *** p < .001).

## References


Aziz, Q., Giamberardino, M. A., Barke, A., Korwisi, B., Baranowski, A. P., Wesselmann, U., . . . Treede, R.-D. (2019). The IASP classification of chronic pain for ICD-11: chronic secondary visceral pain. *Pain*, *160*(1), 69-76.

Bennett, M. I., Kaasa, S., Barke, A., Korwisi, B., Rief, W., & Treede, R.-D. (2019). The IASP classification of chronic pain for ICD-11: chronic cancer-related pain. *Pain*, *160*(1), 38-44.

Benoliel, R., Svensson, P., Evers, S., Wang, S.-J., Barke, A., Korwisi, B., . . . The, I. T. f. t. C. o. C. P. (2019). The IASP classification of chronic pain for ICD-11: chronic secondary headache or orofacial pain. *PAIN*, *160*(1).

Nicholas, M., Vlaeyen, J. W. S., Rief, W., Barke, A., Aziz, Q., Benoliel, R., . . . Treede, R.-D. (2019). The IASP classification of chronic pain for ICD-11: chronic primary pain. *Pain*, *160*(1), 28-37. https://doi.org/10.1097/j.pain.0000000000001390

Perrot, S., Cohen, M., Barke, A., Korwisi, B., Rief, W., & Treede, R.-D. (2019). The IASP classification of chronic pain for ICD-11: chronic secondary musculoskeletal pain. *Pain*, *160*(1), 77-82.

Scholz, J., Finnerup, N. B., Attal, N., Aziz, Q., Baron, R., Bennett, M. I., . . . Treede, R.-D. (2019). The IASP classification of chronic pain for ICD-11: chronic neuropathic pain. *Pain*, *160*(1), 53-59. https://doi.org/10.1097/j.pain.0000000000001365

Schug, S. A., Lavand'homme, P., Barke, A., Korwisi, B., Rief, W., & Treede, R.-D. (2019). The IASP classification of chronic pain for ICD-11: chronic postsurgical or posttraumatic pain. *Pain*, *160*(1), 45-52.

Treede, R.-D., Rief, W., Barke, A., Aziz, Q., Bennett, M. I., Benoliel, R., . . . Wang, S.-J. (2019). Chronic pain as a symptom or a disease: the IASP Classification of Chronic Pain for the International Classification of Diseases (ICD-11). *Pain*, *160*(1), 19-27. https://doi.org/10.1097/j.pain.0000000000001384
